# Associations of prepregnancy body mass index and gestational weight gain with pregnancy outcomes in nulliparous women delivering single live babies

**DOI:** 10.1038/srep12863

**Published:** 2015-08-05

**Authors:** Lu Liu, Zhongxin Hong, Lihong Zhang

**Affiliations:** 1 Department of Nutrition, Beijing Friendship Hospital, Capital Medical University, Beijing 100050, China

## Abstract

The study was to assess the associations of prepregnancy body mass index (BMI) and gestational weight gain (GWG) with pregnancy outcomes. This was a retrospective analysis of 2973 nulliparous women who delivered single live babies. Prepregnancy BMI was categorized as underweight (<18.5 kg/m^2^), normal weight (18.5–24.9 kg/m^2^) or overweight/obese (≥25.0 kg/m^2^). GWG was categorized as inadequate, adequate or excessive. 567 (19.1%) women were overweight/obese, and 1600 (53.8%) exhibited excessive GWG. Compared with women of normal weight, overweight/obese women had a higher incidence of cesarean section (odds ratio, 95% confidence interval: 2.02, 1.59–2.56), postpartum hemorrhage (1.50, 1.05–2.14), preterm delivery (2.51, 1.83–3.45), preterm premature rupture of membranes (2.11, 1.32–3.38), gestational diabetes mellitus (2.04, 1.65–2.53), gestational hypertension (7.68, 4.21–14.00), preeclampsia (1.98, 1.18–3.33) and small for gestational age (2.81, 1.21–6.54). Compared with adequate GWG, excessive GWG increased the incidence of cesarean section (2.02, 1.59–2.56), preterm delivery (1.48, 1.05–2.71), preeclampsia (1.78, 1.34–4.27) and macrosomia (2.61, 1.61–4.25), and reduced the incidence of gestational diabetes mellitus (0.75, 0.62–0.92). High prepregnancy BMI and excessive GWG in nulliparous Chinese women are associated with adverse pregnancy outcomes. Weight control before and during pregnancy could reduce the complications of pregnancy.

The prevalence of maternal obesity has increased over recent years, particularly in developed countries, reflecting the increase of obesity in the general population^1^. This has raised concerns among health professionals, since it is now recognized that a high prepregnancy body mass index (BMI) and excessive or inadequate gestational weight gain (GWG) can have detrimental effects on health outcomes for mother and child, both in the short-term and long-term^1^,^2^,^3^,^4^,^5^. Detailed knowledge of the associations of both prepregnancy BMI and GWG with pregnancy outcomes is essential to determine the scale of the problem and facilitate educational and interventional strategies to minimize the risks of pregnancy.

High prepregnancy BMI and excessive GWG have been reported to impart several risks to the mother, including increased maternal mortality, gestational hypertension (GHT), preeclampsia, gestational diabetes mellitus (GDM) and thromboembolic disorders^1^,^2^,^3^,^4^,^5^. These factors have also been found to influence the outcome of the neonate, with reports of increased perinatal mortality, macrosomia and congenital malformation^1^,^2^,^3^,^4^,^5^. In addition, maternal obesity is associated with more challenging obstetric management, a higher operative delivery rate, and an increased risk of anesthesia. Long-term complications associated with maternal obesity include increased likelihood of maternal weight retention and exacerbation of obesity.

Although several previous studies have examined the influence of high prepregnancy BMI and excessive GWG on perinatal outcomes, relatively few have been conducted in China, and there are only limited data concerning women giving birth in Beijing. To assess the associations of prepregnancy BMI and weight gain during pregnancy with pregnancy outcomes, we collected and analyzed data from 2973 nulliparous women in Beijing, China, who gave birth to single babies. In addition, we also performed a subgroup analysis to explore whether a family history of hypertension or diabetes are associated with adverse pregnancy outcomes, as there is a paucity of relevant data concerning this in the literature.

## Results

### Clinical characteristics of the study participants

A total of 3198 nulliparous women were initially identified for potential inclusion in the study. A total of 225 women were subsequently excluded from the analysis, as follows: incomplete information (*n* = 58); a history of hypertension (*n* = 31), diabetes (*n* = 34), heart disease (*n* = 22), hepatitis (*n* = 28), chronic renal disease (*n* = 23) or other systemic diseases (*n* = 3); drug use (*n* = 7); alcohol use (*n* = 8); and smoking before or during pregnancy (*n* = 11). Therefore, 2973 women were included in the final analysis. The clinical characteristics of these 2973 study participants are presented in Table 1. The median age of the 2973 women at delivery was 29 years (range, 18 to 42 years), the median gestation week at delivery was 39^+4^ weeks (range, 28^+2^ to 41^+5^ weeks), and the median GWG was 15.5 kg.

According to the prepregnancy BMI, 254 women (8.5%) were underweight, 2152 (72.4%) were of normal weight, and 567 (19.1%) were overweight/obese (Table 1). The proportion of women who were overweight/obese was significantly higher in the 30–34 year age group than in younger age groups (*P* < 0.05), and significantly higher in the ≥35 years age group than in younger age groups (*P* < 0.05) (Table 1). A family history of hypertension and a family history of diabetes mellitus were both significantly more prevalent in overweight/obese women than in underweight and normal weight women (*P* < 0.05) (Table 1). In addition, birth weight was significantly higher in overweight/obese women than in normal weight and underweight women (*P* < 0.05) (Table 1). There were no significant differences between the 3 prepregnancy BMI categories in maternal height and gestational weeks (Table 1).

Based on the IOM recommendations for GWG, 404 women (13.6%) exhibited inadequate GWG, 969 (32.6%) exhibited GWG within recommended levels, and 1600 (53.8%) exhibited excessive GWG (Table 1). Prepregnancy BMI was significantly higher in women with excessive GWG (22.6 ± 3.4 kg/m^2^) than in women with adequate (21.9 ± 3.3 kg/m^2^) or inadequate (22.1 ± 2.9 kg/m^2^) GWG (*P* < 0.05) (Table 1). The proportion of women with excessive GWG was significantly higher in the 30–34 years age group than in the 25–29 years age group (*P* < 0.05), and significantly higher in the ≥35 years age group than in younger age groups (*P* < 0.05) (Table 1). A family history of hypertension and a family history of diabetes mellitus were both significantly more prevalent in women with excessive GWG than in women with adequate or inadequate GWG (*P* < 0.05) (Table 1). Gestational weeks and birth weight were both significantly higher in women with excessive GWG than in women with adequate or inadequate GWG (*P* < 0.05) (Table 1). There were no significant differences between the 3 GWG groups in maternal height (Table 1).

Table 2 presents subgroup analysis of data for the various GWG categories according to prepregnancy BMI class. Within each prepregnancy BMI group (underweight, normal weight and overweight/obese), birth weight in the adequate GWG group was significantly higher than that in the inadequate GWG group, and significantly lower than that in the excessive GWG group (with the sole exception that birth weight for overweight/obese women was not significantly different between the adequate and excessive GWG groups). As the prepregnancy BMI increased, the total GWG and mean weight gain per week decreased (*P* < 0.01 for both). In comparison to underweight and normal weight women, overweight/obese women had a lower GWG and were more likely to gain weight above the IOM recommendations (OR, 2.2; 95%CI, 1.3–3.8).

Tables 3 and 4 show the effects of prepregnancy BMI and GWG on pregnancy outcomes, expressed as the odds of each outcome occurring relative to that in women of normal weight or adequate GWG, respectively. In comparison to women of normal weight, overweight/obese women had a higher incidence of cesarean section, PPH, preterm delivery, PPROM, GDM, GHT, preeclampsia and SGA (Table 3), with the association particularly strong for GHT (OR, 7.68; 95%CI, 4.21–14.00). Being underweight was associated with a lower incidence of both GDM and macrosomia. Compared with GWG within the IOM recommendations, excessive GWG increased the incidence of cesarean section, preterm delivery, preeclampsia and infant macrosomia, and reduced the incidence of GDM (Table 4). Inadequate GWG increased the incidence of GDM and SGA.

Table 5 shows the results of a subgroup analysis to determine whether GWG affected pregnancy outcomes in a single prepregnancy BMI category (normal weight women), with the odds of each outcome occurring in the inadequate GWG and excessive GWG groups expressed relative to that in the adequate GWG group. The mean GWG in these 2152 women was 16.0 ± 4.5 kg, with 52.4% (1128/2152) exhibiting excessive GWG. Compared with normal weight women with GWG within the IOM recommendations, normal weight women with excessive GWG showed a higher incidence of cesarean section, PPH, PPROM, GHT and infant macrosomia, and a lower incidence of GDM (Table 5). Inadequate GWG was associated with a higher incidence of preterm delivery, PPROM, GDM and SGA infants, and a lower incidence of cesarean section (Table 5).

Although not a major aim of the present study, additional subgroup analyses were performed to explore the associations of a family history of hypertension and a family history of diabetes mellitus with the clinical characteristics and pregnancy outcomes of the study participants (Table 6), as relatively few studies have examined these associations. Women with either a family history of hypertension or a family history of diabetes mellitus exhibited significantly older mean age, higher prepregnancy BMI with a larger proportion in the overweight/obese category, more excessive GWG, increased incidence of PPH and GDM, and decreased incidence of preterm delivery and PPROM (Table 6). In addition, women with a family history of hypertension had a longer gestation, higher infant birth weight and increased incidence of GHT, while those with a family history of diabetes mellitus had a lower infant birth weight and increased incidence of SGA and macrosomia (Table 6). Logistic regression analysis indicated that PPH (OR, 1.80; 95%CI, 1.18–2.73; *P* = 0.006), preterm birth (OR, 0.20; 95%CI, 0.08–0.46; *P* < 0.001), SGA (OR, 3.52; 95%CI, 1.53–8.10; *P* = 0.003) and macrosomia (OR, 1.51; 95%CI, 1.05–2.18; *P* = 0.028) were all associated with a family history of diabetes mellitus, while preterm birth (OR, 0.42; 95%CI, 0.22–0.80; *P* = 0.008) was associated with a family history of hypertension.

## Discussion

The main findings of the present study are that, compared to women of normal weight, overweight/obese women had a higher incidence of cesarean section, PPH, preterm delivery, PPROM, GDM, GHT, preeclampsia and SGA, while underweight women had a lower incidence of both GDM and macrosomia. Furthermore, compared with GWG within the IOM recommendations, excessive GWG increased the incidence of cesarean section, preterm delivery, preeclampsia and infant macrosomia, and reduced the incidence of GDM, while inadequate GWG increased the incidence of GDM and SGA. In addition, PPH, preterm birth, SGA and macrosomia were all associated with a family history of diabetes mellitus, while preterm birth was associated with a family history of hypertension. These data in a cohort of women at a hospital in Beijing (China) not only corroborate the findings of several previous studies in other regions (of China and other countries), but also provide novel insights regarding the possible influences of prepregnancy BMI, GWG, and family history of hypertension or diabetes on pregnancy outcomes.

In this retrospective analysis, 19.1% of women delivering single live babies in Beijing were overweight or obese. A previous study in Sweden^6^ found that the prevalence of overweight women (BMI ≥ 25 kg/m^2^) was 32.8%, possibly reflecting a higher incidence of maternal obesity in Europe. In our study, total GWG decreased with increasing prepregnancy BMI. Nonetheless, although total GWG was lower in overweight/obese women, the proportion of women with a GWG that exceeded the IOM recommendations was higher in overweight/obese women than in women of normal weight (66.7% *vs.* 52.4%). These observations are consistent with those of a study in a Hispanic population^7^, which demonstrated that women in the highest BMI category gained less weight than those in the lowest category. An additional finding of the present study was that the odds of excessive GWG were higher in overweight women than in women of normal weight. A possible reason for this may be the lower recommended GWG for overweight/obese women in the IOM guidelines.

The odds of cesarean section were increased in both overweight/obese women (OR ~1.5) and in women with excessive GWG (OR ~2.0), in agreement with several previous investigations. For example, Young *et al.*^8^ found that overweight women (BMI 25–30 kg/m^2^) had 2.6-fold increased odds for cesarean section compared with underweight women (BMI < 20 kg/m^2^), while a study in China^9^ showed that cesarean section was more common in overweight and obese women regardless of the time of labor. In the present study, a subgroup analysis of women with normal prepregnancy BMI revealed that the proportion of women undergoing cesarean section was higher in those with excessive GWG and lower in those with inadequate GWG. Therefore, there was a clear association between GWG and frequency of cesarean section in women with a normal prepregnancy BMI.

Numerous previous investigations have explored the risk factors for PPH. For example, in a study of 11363 nulliparous women, Fyfe *et al.*^10^ determined that the odds of PPH were approximately two times higher in obese women than in women of normal weight (OR, 1.83; 95%CI, 1.51–2.28). Jain *et al.*^11^ reported an even stronger influence of obesity, with the odds of PPH more than 5 times higher in obese women than in women with a normal BMI (OR, 5.11; 95%CI, 1.76–14.79). Our finding of an increased frequency of PPH in overweight/obese women (OR ~1.5) was in agreement with these previous reports. Xu *et al.*^12^ determined that the odds of PPH in women with excessive GWG were double those in women with normal GWG. In our study, there was no significant association between GWG category and PPH after adjustment for birth weight and prepregnancy BMI. However, in women with a prepregnancy BMI within the normal range, excessive GWG was associated with a higher incidence of PPH (OR ~2.0 relative to women with adequate GWG). Thus, excessive GWG appears to be associated with PPH in women of normal weight, suggesting that the influence of GWG is independent of any effects of maternal prepregnancy weight.

In our study, the ORs for preterm delivery and PPROM were not adjusted for metabolic syndromes of pregnancy such as GDM, GHT and preeclampsia, even though these diseases are associated with increased systemic inflammation and are considered important risk factors for preterm delivery and PPROM. It was not considered appropriate to adjust for these factors because it was possible that they might act as potential mediators in the relationship between maternal weight/GWG and preterm delivery/PPROM. The odds of preterm delivery were increased in both overweight/obese women (OR ~2.5) and women with excessive GWG (OR ~1.5), whereas PPROM was associated only with the overweight/obese category (OR ~2.1). These findings are consistent with those of Nohr *et al.*^13^, who reported that the adjusted ORs for preterm delivery and PPROM in women with a BMI ≥ 30 kg/m^2^ (compared with women of normal weight) were 1.2 and 1.5, respectively. Chen *et al.*^14^ have also found that high weight gain in pregnancy was associated with an increased occurrence of PPROM.

Previous research in China has determined that the prevalence of GDM has increased from 2.4% in 1999 to 6.8% in 2008. This increase has been attributed to changes in diet (i.e., a higher-energy and higher-fat diet), increased prevalence of maternal obesity and high GWG, and more stringent diagnostic criteria^15^. In agreement with other studies, we found the incidence of GDM to be higher in women who were overweight/obese before pregnancy, and lower in women who were underweight before pregnancy. In a study by Abenhaim *et al.*^16^, women with a high prepregnancy BMI had approximately twice the odds of suffering GDM. In contrast to other studies^17^, we found a strong negative association between GWG and GDM. However, this finding does not necessarily imply that a high GWG in itself decreased the incidence of GDM. The reason for this is that almost all the women with GDM received intervention from a dietician, which involved strict control of weight gain during the third trimester. We speculate that weight gain during the first and second trimesters could be better predictors of GDM occurrence, although we did not have the data at our disposal to test this hypothesis.

Our study showed that the odds of GHT and preeclampsia were much higher in women who were overweight or obese before pregnancy (ORs ~7.7 and ~2.0, respectively). Furthermore, the odds of preeclampsia were also higher in women with GWG above the IOM recommendations (OR ~1.8). Our observations concur with data from several previous investigations. Gaillard *et al.*^18^ determined the odds of GHT and preeclampsia to be 3-fold and 2-fold higher in women with a high prepregnancy BMI. Fortner *et al.*^19^ found that Latina women with a high GWG had approximately 3 times the odds of experiencing GHT and 4 times the odds of experiencing preeclampsia, compared to women with normal GWG. Jensen *et al.*^20^ reported that increased GWG was strongly associated with GHT (OR, 4.8; 95%CI, 1.7–13.1). Although our study showed no significant association between GWG and GHT overall, it is notable that a strong association was evident in women with a normal prepregnancy BMI (OR ~3.4).

After adjustments were made for possible confounding factors, the odds of SGA were calculated to be higher in women who were overweight or obese before pregnancy and in those who gained less weight during pregnancy. In addition, the odds of macrosomia were reduced in underweight women (OR ~0.5), and increased in women with excessive GWG (OR ~2.6). Our observations are in keeping with previous studies reporting associations between GWG and infant birth weight^21^,^22^,^23^.

Few studies have examined the relationships between family history of diabetes or hypertension and perinatal outcomes. In the present study, PPH, preterm birth, SGA and macrosomia were all associated with a family history of diabetes mellitus, while preterm birth was associated with a family history of hypertension. Previous investigations have reported that a family history of hypertension is associated with preeclampsia^24^,^25^,^26^, and that a family history of diabetes mellitus is associated with GDM^27^,^28^. However, further studies are merited to better define the relevance of family history of hypertension or diabetes to the various adverse outcomes of pregnancy.

This study has some limitations. First, this was an observational, retrospective, single-center study; hence selection and information bias cannot be excluded, and the enrolled cohort may not be representative of the general population in China or beyond. Second, prepregnancy BMI was calculated from self-reported prepregnancy weight and height values, which may have led to reporting bias. Third, there was no assessment of participant diet and physical activity. Diet and physical activity may have important influences on GWG, thus it was not possible to discriminate the associations between maternal weight/GWG and perinatal outcomes from any associations between diet/physical activity and these perinatal outcomes. Fourth, the relevance of the IOM guidelines for categorizing GWG in Chinese women has yet to be established. Fifth, it was not possible to explore the relative importance of GWG in each trimester of pregnancy to perinatal outcomes, as relevant data were unavailable. Sixth, the association between GWG and metabolic syndromes of pregnancy remain unclear. Seventh, women with GDM received intervention during the third trimester (dietary control of energy intake plus insulin therapy if required), which may have influenced the associations between GWG and GDM. Finally, our study did not make a distinction between overweight and obese categories, which may have missed or decreased the strength of associations of prepregnancy weight with certain perinatal outcomes.

In conclusion, high prepregnancy BMI and excessive GWG are associated with higher incidences of cesarean section, PPH, preterm delivery, PPROM, GDM, GHT, preeclampsia, SGA and macrosomia in nulliparous women delivering a single baby in Beijing, China. Knowledge of this information can assist in the design and implementation of educational and interventional strategies to minimize the risks of pregnancy.

## Methods

### Study participants

The present study was a retrospective analysis of data collected prospectively from consecutive nulliparous women who delivered single live babies at the Beijing Friendship Hospital affiliated to Capital Medical University (Beijing, China) between October 2013 and October 2014. The exclusion criteria were as follows: an incomplete dataset available; a history of hypertension, diabetes, heart disease, hepatitis, chronic renal disease or other systemic disease; a history of drug use before or during pregnancy; a history of alcohol use before or during pregnancy; or a history of smoking before or during pregnancy. The study was approved by the ethics committee and institutional review board of Beijing Friendship Hospital, and all participants provided informed written consent.

### Clinical data collection

The following clinical characteristics were obtained from the medical records: maternal age; family history of diabetes or hypertension; maternal prepregnancy weight and height; maternal weight before delivery; gestational age at delivery; mode of delivery; birth weight of the baby; maternal complications, including postpartum hemorrhage (PPH), preterm birth, preterm premature rupture of the membranes (PPROM), GDM, GHT and preeclampsia; and neonatal complications, including small for gestational age (SGA) and macrosomia.

### Prepregnancy BMI and GWG

Prepregnancy BMI was calculated as weight (kg) divided by height (m) squared, using self-reported information on prepregnancy weight and height provided during the first prenatal care visit. Prepregnancy BMI was categorized as either underweight (<18.5 kg/m^2^), normal weight (18.5–24.9 kg/m^2^) or overweight/obese (≥25.0 kg/m^2^), according to World Health Organization (WHO) criteria^29^. Maternal GWG (weight before delivery minus prepregnancy weight) was categorized according to Institute of Medicine (IOM) recommendations^30^, as follows. Inadequate GWG was defined as a weight gain during pregnancy of <12.5 kg in underweight women (BMI < 18.5 kg/m^2^), <11.5 kg in normal weight women (BMI 18.5–24.9 kg/m^2^), <7 kg in overweight women (BMI 25.0–30.0 kg/m^2^), and <5 kg in obese women (≥30.0 kg/m^2^). Excessive GWG was defined as a weight gain during pregnancy of >18 kg in underweight women (BMI < 18.5 kg/m^2^), >16 kg in normal weight women (BMI 18.5–24.9 kg/m^2^), >11.5 kg in overweight women (BMI 25.0–30.0 kg/m^2^), and >9 kg in obese women (≥30.0 kg/m^2^). All others were classified as adequate GWG (i.e. within the IOM recommendations). The mean weight gain per week during pregnancy was calculated as total GWG divided by the number of gestational weeks.

### Maternal outcomes

Cesarean deliveries included prelabor elective cesarean section delivery, prelabor emergency cesarean section delivery, and emergency cesarean section delivery in labor. PPH was defined as blood loss ≥500 mL within 24 hours of delivery, as recorded on the delivery summary by a nurse or doctor, regardless of the mode of delivery. Preterm birth was defined as the delivery of a live infant before 37 weeks of gestation. PPROM was defined as a spontaneous rupture of membranes before the onset of labor and before 37 weeks of gestation. GDM was defined in accordance with International Association of Diabetic Pregnancy Study Group (IADPSG) criteria^31^. GHT was defined as the development of arterial hypertension (as a new phenomenon) in a pregnant woman after 20 weeks of gestation, without enough proteinuria, symptoms or laboratory abnormalities to be classified as preeclampsia. Preeclampsia was defined as the combination of hypertension and proteinuria (≥300 mg of protein in a 24-hour urine sample) occurring after 20 weeks of gestation. A hypertensive disorder of pregnancy (i.e., GHT and preeclampsia) at any prenatal visit was considered a severe clinical condition.

### Neonatal outcomes

SGA was defined as a birth weight <2500 g in a baby delivered after 37 weeks of gestation. Macrosomia was defined as birth weight ≥4000 g.

### Statistical analysis

Statistical analyses were performed using SPSS 17.0 (SPSS Inc., Chicago, IL, USA). Data are expressed as the mean ± standard deviation, median (range) or number (percentage). Tests of normality indicated that all continuous data in the current study were normally distributed; therefore, statistical comparisons were made using analysis of variance (ANOVA) and a Student-Newman-Keuls post-hoc test. Statistical comparisons of categorical data were made using the chi-squared (χ^2^) test. A *P* value < 0.05 was considered statistically significant.

Multivariate logistic regression analysis was performed to identify associations between prepregnancy BMI or GWG and various pregnancy outcomes (cesarean section, PPH, preterm birth, PPROM, GDM, GHT, preeclampsia, SGA and macrosomia), after adjustment for potential confounding variables. Adjusted odds ratios (ORs) and 95% confidence intervals (CIs) were calculated for each pregnancy outcome, in order to determine the odds of high or low prepregnancy BMI relative to normal prepregnancy BMI, and of inadequate or excessive GWG relative to adequate GWG. All ORs were adjusted for maternal age, maternal height, family history of hypertension, family history of diabetes, and prepregnancy BMI or GWG (as appropriate). Additional adjustments were made, as follows: gestational weeks and birth weight for cesarean section and PPH; and gestational weeks for GDM, GHT, preeclampsia, SGA and macrosomia.

## Additional Information

**How to cite this article**: Liu, L. *et al.* Associations of prepregnancy body mass index and gestational weight gain with pregnancy outcomes in nulliparous women delivering single live babies. *Sci. Rep.* **5**, 12863; doi: 10.1038/srep12863 (2015).

## Figures and Tables

**Table 1:** Clinical characteristics of the study participants grouped by prepregnancy body mass index and gestational weight gain.

	Prepregnancy BMI (kg/m^2^)			GWG categories by IOM guidelines (kg)		
	Underweight	Normal weight	Overweight/obese	Below	Within	Above
*N* (%)	254 (8.5)	2152 (72.4)	567 (19.1)	404 (13.6)	969 (32.6)	1600 (53.8)
Age, years						
<25	18 (12.6)	107 (74.8)	18 (12.6)	20 (14.0)	40 (28.0)	83 (58.0)
25–29	154 (10.8)	1031 (72.7)	234 (16.5)	170 (12.0)	438 (30.9)	811 (57.1)
30–34	70 (5.6)	909 (73.0)	267 (21.4)^a^	180 (14.4)	421 (33.8)	645 (51.8)^1^
≥35	12 (7.3)	105 (63.6)	48 (29.1)^b^	34 (20.6)	70 (42.4)	61 (37.0)^2^
Height, cm						
<160	82 (9.8)	605 (72.0)	153 (18.2)	116 (13.8)	283 (33.7)	441 (52.5)
160–164	106 (8.2)	936 (72.2)	255 (19.6)	184 (14.2)	420 (32.4)	693 (53.4)
>165	66 (7.9)	611 (73.1)	159 (19.0)	104 (12.5)	266 (31.8)	466 (55.7)
Family history of hypertension	26 (10.2)	175 (8.1)	135 (23.8)^c^	33 (8.2)	83 (8.6)	220 (13.8)^3^
Family history of diabetes	16 (6.3)	171 (7.9)	108 (19.0)^d^	41 (10.1)	68 (7.0)	186 (11.6)^4^
Gestational weeks, weeks	39.3 ± 1.6	39.2 ± 1.7	39.4 ± 1.8	38.7 ± 2.1^5^	39.2 ± 1.6^5^	39.4 ± 1.6^5^
Birth weight, g	3266 ± 420^e^	3356 ± 522^e^	3480 ± 484^e^	3151 ± 566^6^	3299 ± 516^6^	3472 ± 465^6^
Prepregnancy BMI, kg/m^2^				22.1 ± 2.9	21.9 ± 3.3	22.6 ± 3.4^7^

Data presented as *n* (%) or mean ± standard deviation. BMI, body mass index; GWG, gestational weight gain; IOM, Institute of Medicine. ^a^*P* < 0.05 *vs.* both younger age groups; ^b^*P* < 0.05 *vs.* all 3 younger age groups; ^c^*P* < 0.05 *vs.* underweight and normal weight groups; ^d^*P* < 0.05 *vs.* underweight and normal weight groups; ^e^*P* < 0.05 *vs.* both the other two prepregnancy BMI groups; ^1^*P* < 0.05 *vs.* 25–29 year age group; ^2^*P* < 0.05 *vs.* all three younger age groups; ^3^*P* < 0.05 *vs.* inadequate (‘below’) and normal (‘within’) GWG groups; ^4^*P* < 0.05 *vs.* normal (‘within’) GWG group; ^5^*P* < 0.05 *vs.* both the other two GWG groups; ^6^*P* < 0.05 *vs.* both the other two GWG groups; ^7^*P* < 0.0^5^ *vs.* both the other two GWG groups.

**Table 2:** Variation of birth weight, total GWG and mean weight gain per week with GWG category in each of the three prepregnancy BMI groups.

BMI	GWG category according to IOM	*N* (%)	Birth weight (g)	Total gestational weight gain (kg)	Mean weight gain per week (kg)
Underweight	Inadequate	46 (18.1)	2928 ± 455*	10.7 ± 1.7*	0.28 ± 0.05*
	Adequate	114 (44.9)	3271 ± 368*	14.9 ± 1.4*	0.38 ± 0.04*
	Excessive	94 (37.0)	3426 ± 366*	20.7 ± 3.8*	0.52 ± 0.09*
Normal weight	Inadequate	307 (14.3)	3169 ± 526*	9.0 ± 1.8*	0.23 ± 0.05*
	Adequate	717 (33.3)	3279 ± 543*	13.8 ± 1.2*	0.35 ± 0.03*
	Excessive	1128 (52.4)	3456 ± 485*	19.3 ± 3.0*	0.49 ± 0.08*
Overweight	Inadequate	51 (9.0)	3237 ± 587*	4.4 ± 1.7*	0.12 ± 0.05*
	Adequate	138 (24.3)	3424 ± 457†	9.0 ± 1.7*	0.23 ± 0.04*
	Excessive	378 (66.7)	3532 ± 421†	16.4 ± 54.2*	0.41 ± 0.11*

Data presented as *n* (%) or mean ± standard deviation. BMI, body mass index; GWG, gestational weight gain; IOM, Institute of Medicine. *P < 0.05 *vs.* both the other GWG groups; †*P* < 0.05 *vs.* inadequate GWG group.

**Table 3:** Effects of prepregnancy body mass index on pregnancy outcomes.

	Underweight (*N* = 254)			Normal weight (*N* = 2152)	Overweight/obese (*N* = 567)		
	*N* (%)	*P*	AOR (95%CI)	*N* (%)	*N* (%)	*P*	AOR (95%CI)
Cesarean section^a^	98 (38.6)	0.265	0.86 (0.65–1.13)	1028 (47.8)	348 (61.4)	0.000	1.52 (1.24–1.86)
PPH^a^	6 (2.4)	0.078	0.47 (0.20–1.09)	117 (5.4)	53 (9.3)	0.027	1.50 (1.05–2.14)
Preterm delivery^b^	11 (4.3)	0.115	0.69 (0.32–1.13)	143 (6.6)	69 (12.2)	0.000	2.51 (1.83–3.45)
PPROM^b^	4 (1.6)	0.179	0.60 (0.18–1.38)	65 (3.0)	28 (4.9)	0.002	2.11 (1.32–3.38)
GDM^c^	38 (15.0)	0.026	0.66 (0.46–0.95)	453 (21.1)	201 (35.4)	0.000	2.04 (1.65–2.53)
GHT^c^	5 (2.0)	0.195	1.95 (0.71–5.32)	19 (0.9)	33 (5.8)	0.000	7.68 (4.21–14.00)
Preeclampsia^c^	4 (1.6)	0.164	0.47 (0.16–1.37)	60 (2.8)	26 (4.6)	0.010	1.98 (1.18–3.33)
SGA^c^	4 (1.6)	0.241	1.97 (0.63–6.11)	15 (0.7)	11 (1.9)	0.017	2.81 (1.21–6.54)
Macrosomia^c^	15 (5.9)	0.024	0.53 (0.30–0.92)	247 (11.5)	66 (11.6)	0.140	0.79 (0.58–1.08)

AOR, adjusted odds ratio; CI, confidence interval; GDM, gestational diabetes mellitus; GHT, gestational hypertension; PPH, postpartum hemorrhage; PPROM, preterm premature rupture of membranes; SGA, small for gestational age. AORs are presented relative to the normal weight group; ^a^adjusted for maternal age, height, gestational weeks, family history of hypertension, family history of diabetes, gestational weight gain and birth weight; ^b^adjusted for maternal age, height, family history of hypertension, family history of diabetes and gestational weight gain; ^c^adjusted for maternal age, height, gestational weeks, family history of hypertension, family history of diabetes and gestational weight gain.

**Table 4:** Effects of gestational weight gain on pregnancy outcomes.

	Inadequate GWG (*N* = 404)			Adequate GWG (*N* = 969)	Excessive GWG (*N* = 1600)		
	*N* (%)	*P*	AOR (95%CI)	*N* (%)	*N* (%)	*P*	AOR (95%CI)
Cesarean section^a^	150 (37.1)	0.145	0.84 (0.66–1.07)	412 (42.5)	912 (57.0)	0.000	2.02 (1.59–2.56)
PPH^a^	23 (5.7)	0.141	1.53 (0.90–2.62)	41 (4.2)	112 (7.0)	0.576	1.31 (0.89–1.91)
Preterm delivery^b^	35 (8.7)	0.293	1.12 (0.86–1.67)	55 (5.7)	133 (8.3)	0.040	1.48 (1.05–2.71)
PPROM^b^	21 (5.2)	0.074	1.60 (0.64–2.75)	31 (3.2)	45 (2.8)	0.054	0.51 (0.30–1.17)
GDM^c^	151 (37.4)	0.000	2.11 (1.63–2.74)	224 (23.1)	317 (19.8)	0.011	0.75 (0.62–0.92)
GHT^c^	8 (2.0)	0.926	0.96 (0.41–2.24)	20 (2.1)	29 (1.8)	0.216	0.59 (0.26–1.36)
Preeclampsia^c^	7 (1.7)	0.185	0.52 (0.09–2.04)	22 (2.3)	61 (3.8)	0.010	1.78 (1.34–4.27)
SGA^c^	9 (2.2)	0.000	3.45 (1.28–9.80)	8 (0.8)	13 (0.8)	0.202	0.92 (0.52–1.04)
Macrosomia^c^	20 (5.0)	0.325	1.30 (0.77–2.20)	71 (7.3)	237 (14.8)	0.000	2.61 (1.61–4.25)

AOR, adjusted odds ratio; BMI, body mass index; CI, confidence interval; GDM, gestational diabetes mellitus; GHT, gestational hypertension; GWG, gestational weight gain; PPH, postpartum hemorrhage; PPROM, preterm premature rupture of membranes; SGA, small for gestational age. AORs are presented relative to the adequate GWG group; ^a^adjusted for maternal age, height, gestational weeks, family history of hypertension, family history of diabetes, prepregnancy body mass index and birth weight; ^b^adjusted for maternal age, height, family history of hypertension, family history of diabetes and prepregnancy body mass index; ^c^adjusted for maternal age, height, gestational weeks, family history of hypertension, family history of diabetes and prepregnancy body mass index.

**Table 5:** Effects of gestational weight gain on pregnancy outcomes in women of normal weight (*N* = 2152).

	Inadequate GWG (*N* = 307)			Adequate GWG (*N* = 717)	Excessive GWG (*N* = 1128)		
	*N* (%)	*P*	AOR (95%CI)	*N* (%)	*N* (%)	*P*	AOR (95%CI)
Cesarean section^a^	101 (32.9)	0.006	0.67 (0.50–0.89)	300 (41.8)	627 (55.6)	0.000	1.71 (1.41–2.09)
PPH^a^	12 (3.9)	0.843	0.93 (0.46–1.89)	27 (3.8)	78 (6.9)	0.000	1.97 (1.25–3.13)
Preterm delivery^b^	29 (9.4)	0.048	1.65 (1.01–2.72)	49 (6.8)	65 (5.8)	0.432	0.86 (0.58–1.26)
PPROM^b^	18 (5.9)	0.000	5.00 (2.21–11.28)	18 (1.6)	29 (4.0)	0.008	3.30 (1.26–8.65)
GDM^c^	114 (37.1)	0.000	2.04 (1.51–2.75)	159 (22.2)	180 (16.0)	0.001	0.67 (0.52–0.85)
GHT^c^	1 (0.3)	0.741	0.70 (0.08–5.92)	6 (0.5)	12 (1.7)	0.017	3.37 (1.24–9.17)
Preeclampsia^c^	3 (1.0)	0.087	0.32 (0.09–1.18)	18 (2.5)	39 (3.5)	0.143	1.58 (0.86–2.90)
SGA^c^	6 (2.0)	0.020	2.84 (1.12–9.05)	6 (0.8)	3 (0.3)	0.118	0.33 (0.08–1.33)
Macrosomia^c^	13 (4.2)	0.137	0.61 (0.32–1.17)	51 (7.1)	183 (16.2)	0.000	2.23 (1.59–3.14)

AOR, adjusted odds ratio; BMI, body mass index; CI, confidence interval; GDM, gestational diabetes mellitus; GHT, gestational hypertension; GWG, gestational weight gain; PPH, postpartum hemorrhage; PPROM, preterm premature rupture of membranes; SGA, small for gestational age. AORs are presented relative to the adequate GWG group; ^a^adjusted for maternal age, height, gestational weeks, family history of hypertension, family history of diabetes, prepregnancy body mass index and birth weight; ^b^adjusted for maternal age, height, family history of hypertension, family history of diabetes and prepregnancy body mass index; ^c^adjusted for maternal age, height, gestational weeks, family history of hypertension, family history of diabetes and prepregnancy body mass index.

**Table 6:** Clinical characteristics of the study participants grouped by family history of hypertension and family history of diabetes mellitus.

	Family history of hypertension			Family history of diabetes mellitus		
	Yes	No	*P*	Yes	No	*P*
*N* (%)	336	2637		295	2678	
Age, years	29.7 ± 3.2	29.3 ± 3.2	0.023	29.9 ± 2.7	29.3 ± 3.2	0.001
<25	15 (4.5)	128 (4.9)		6 (2.0)	137 (5.1)	
25–29	150 (44.6)	1269 (48.1)		116 (39.3)	1303 (48.7)	
30–34	149 (44.3)	1097 (41.6)		164 (55.6)	1082 (40.4)	
≥35	22 (6.5)	143 (5.4)		9 (3.1)	156 (5.8)	
Height, cm	163.1 ± 5.0	163.0 ± 4.7	0.879	163.1 ± 4.7	163.0 ± 4.7	0.865
Gestational weeks, weeks	39.5 ± 1.6	39.2 ± 1.7	0.007	39.4 ± 1.3	39.2 ± 1.7	0.172
Birth weight, g	3474.0 ± 454.9	3359.0 ± 515.5	0.000	3455.4 ± 417.2	3362.8 ± 518.7	0.003
Prepregnancy BMI, kg/m^2^	23.7 ± 3.9	22.1 ± 3.2	0.000	23.9 ± 4.3	22.1 ± 3.2	0.000
Underweight	26 (7.7)	228 (8.6)		16(5.4)	238 (8.9)	
Normal weight	175 (52.1)	1977 (75)		171(58.0)	1981 (74.0)	
Overweight/obese	135 (40.2)	432 (16.4)		108(36.6)	459 (17.1)	
GWG, kg	15.3 ± 4.2	15.6 ± 4.9	0.246	14.6 ± 4.2	15.7 ± 4.8	0.000
Inadequate	33 (9.8)	371 (14.1)		41 (13.9)	363 (13.6)	
Adequate	83 (24.7)	886 (33.6)		68 (23.1)	901 (33.6)	
Excessive	220 (65.5)	1380 (52.3)		186 (63.1)	1414 (52.8)	
Cesarean section	174/162 (51.8)	1300/1337 (49.3)	0.390	153/142 (51.9)	1321/1357 (49.3)	0.408
PPH	31/305 (9.2)	145/2492 (5.5)	0.006	32/263 (10.8)	144/2534 (5.4)	0.000
Preterm delivery	11/325 (3.3)	212/2425 (8.0)	0.002	6/289 (2.0)	217/2461 (8.1)	0.000
PPROM	4/332 (1.2)	93/2544 (3.5)	0.023	3/292 (1.0)	92/2586 (3.6)	0.025
GDM	94/242 (28.0)	598/2039 (22.7)	0.030	87/208 (29.5)	605/2073 (22.6)	0.008
GHT	12/324 (3.6)	45/2592 (1.7)	0.019	3/292 (1.0)	54/2624 (2.0)	0.235
Preeclampsia	12/324 (3.6)	78/2559 (3.0)	0.537	4/291 (1.4)	86/2592 (3.2)	0.078
SGA	6/330 (1.8)	24/2613 (0.9)	0.130	9/286 (3.1)	21/2657 (0.8)	0.000
Macrosomia	43/293 (12.8)	285/2352 (10.8)	0.273	45/250 (15.3)	283/2395 (10.6)	0.015

Data presented as *n* (%) or mean ± standard deviation. BMI, body mass index; GDM, gestational diabetes mellitus; GHT, gestational hypertension; GWG, gestational weight gain; PPH, postpartum hemorrhage; PPROM, preterm premature rupture of membranes; SGA, small for gestational age.
